# Supplementary material for: Development and Application of Potentially Universal Microsatellite Markers for Pheasant Species
Source: Animals (Basel). 2023 Nov 21;13(23):3601. doi: 10.3390/ani13233601 (PMC10705216; doi:10.3390/ani13233601)
Supplement: Supplementary file 1 [file animals-13-03601-s001.zip › Supplementary File S1.doc]

**Table S1** Statistics of blood and tissue sample collection and DNA extraction of pheasant

| Number | Name of species | Scientific Name | Location of collection | Gender | Date of collection | Sample Type | Result of DNA extraction |
| --- | --- | --- | --- | --- | --- | --- | --- |
| SCGB00024 | Blood Pheasant | *Ithaginis cruentus* | Pamuling, Yajiang County, Sichuan Province | ♀ | 2006.5.2 | Muscle | × |
| SCGB00208 | Blood Pheasant | *Ithaginis cruentus* | Pamuling, Yajiang County, Sichuan Province | ♀ | 2008.11.16 | Blood | √ |
| SCGB00209 | Blood Pheasant | *Ithaginis cruentus* | Pamuling, Yajiang County, Sichuan Province | ♂ | 2008.11.16 | Blood | √ |
| SCGB00210 | Blood Pheasant | *Ithaginis cruentus* | Pamuling, Yajiang County, Sichuan Province | ♂ | 2008.11.16 | Blood | × |
| SCGB00240 | Blood Pheasant | *Ithaginis cruentus* | Pamuling, Yajiang County, Sichuan Province | ♂ | 2009.3.15 | Blood | × |
| SCGB00241 | Blood Pheasant | *Ithaginis cruentus* | Pamuling, Yajiang County, Sichuan Province | ♂ | 2009.3.15 | Blood | × |
| SCGB00242 | Blood Pheasant | *Ithaginis cruentus* | Pamuling, Yajiang County, Sichuan Province | ♂ | 2009.3.15 | Blood | √ |
| SCGB00243 | Blood Pheasant | *Ithaginis cruentus* | Pamuling, Yajiang County, Sichuan Province | ♂ | 2009.3.15 | Blood | √ |
| SCGB00244 | Blood Pheasant | *Ithaginis cruentus* | Pamuling, Yajiang County, Sichuan Province | ♂ | 2009.3.15 | Blood | √ |
| SCGB00245 | Blood Pheasant | *Ithaginis cruentus* | Pamuling, Yajiang County, Sichuan Province | ♂ | 2009.3.15 | Blood | × |
| SCGB00246 | Blood Pheasant | *Ithaginis cruentus* | Pamuling, Yajiang County, Sichuan Province | ♂ | 2009.3.15 | Blood | √ |
| SCGB00247 | Blood Pheasant | *Ithaginis cruentus* | Pamuling, Yajiang County, Sichuan Province | ♀ | 2009.3.15 | Blood | × |
| SCGB00248 | Blood Pheasant | *Ithaginis cruentus* | Pamuling, Yajiang County, Sichuan Province | ♂ | 2009.3.15 | Blood | √ |
| SCGB00249 | Blood Pheasant | *Ithaginis cruentus* | Pamuling, Yajiang County, Sichuan Province | ♀ | 2009.3.15 | Blood | √ |
| SCGB00250 | Blood Pheasant | *Ithaginis cruentus* | Pamuling, Yajiang County, Sichuan Province | ♀ | 2009.3.15 | Blood | √ |
| SCGB00251 | Blood Pheasant | *Ithaginis cruentus* | Pamuling, Yajiang County, Sichuan Province | ♀ | 2009.3.15 | Blood | √ |
| SCGB00252 | Blood Pheasant | *Ithaginis cruentus* | Pamuling, Yajiang County, Sichuan Province | ♂ | 2009.3.15 | Blood | √ |
| SCGB00253 | Blood Pheasant | *Ithaginis cruentus* | Pamuling, Yajiang County, Sichuan Province | ♂ | 2009.3.15 | Blood | √ |
| SCGB00254 | Blood Pheasant | *Ithaginis cruentus* | Pamuling, Yajiang County, Sichuan Province | ♂ | 2009.3.15 | Blood | √ |
| SCGB00255 | Blood Pheasant | *Ithaginis cruentus* | Pamuling, Yajiang County, Sichuan Province | ♂ | 2009.3.15 | Blood | √ |
| SCGB00256 | Blood Pheasant | *Ithaginis cruentus* | Pamuling, Yajiang County, Sichuan Province | ♂ | 2009.4.17 | Blood | √ |
| SCGB00257 | Blood Pheasant | *Ithaginis cruentus* | Pamuling, Yajiang County, Sichuan Province | ♂ | 2009.4.17 | Blood | √ |
| SCGB00258 | Blood Pheasant | *Ithaginis cruentus* | Pamuling, Yajiang County, Sichuan Province | ♂ | 2009.4.17 | Blood | √ |
| SCGB00259 | Blood Pheasant | *Ithaginis cruentus* | Pamuling, Yajiang County, Sichuan Province | ♂ | 2009.4.17 | Blood | √ |
| SCGB00260 | Blood Pheasant | *Ithaginis cruentus* | Pamuling, Yajiang County, Sichuan Province | ♀ | 2009.4.17 | Blood | √ |
| SCGB00261 | Blood Pheasant | *Ithaginis cruentus* | Pamuling, Yajiang County, Sichuan Province | ♂ | 2009.3.15 | whole | × |
| SCGB00278 | Blood Pheasant | *Ithaginis cruentus* | Pamuling, Yajiang County, Sichuan Province | ♂ | 2009.4.17 | digestive system | × |
| SCGB00279 | Blood Pheasant | *Ithaginis cruentus* | Pamuling, Yajiang County, Sichuan Province | ♂ | 2009.4.17 | Muscle | × |
| SCGB00058 | Lady Amherst’s Pheasant | *Chrysolophus amherstiae* | Muping Town, Baoxing County, Sichuan Province | -- | 2007.12.25 | Muscle | × |
| SCGB00059 | Lady Amherst’s Pheasant | *Chrysolophus amherstiae* | Muping Town, Baoxing County, Sichuan Province | -- | 2007.12.25 | Muscle | × |
| SCGB00060 | Lady Amherst’s Pheasant | *Chrysolophus amherstiae* | Muping Town, Baoxing County, Sichuan Province | -- | 2007.12.25 | Muscle | √ |
| SCGB00061 | Lady Amherst’s Pheasant | *Chrysolophus amherstiae* | Muping Town, Baoxing County, Sichuan Province | -- | 2007.12.25 | Muscle | √ |
| SCGB00062 | Lady Amherst’s Pheasant | *Chrysolophus amherstiae* | Muping Town, Baoxing County, Sichuan Province | -- | 2007.12.25 | Muscle | × |
| SCGB00063 | Lady Amherst’s Pheasant | *Chrysolophus amherstiae* | Muping Town, Baoxing County, Sichuan Province | -- | 2007.12.25 | Muscle | × |
| SCGB00064 | Lady Amherst’s Pheasant | *Chrysolophus amherstiae* | Muping Town, Baoxing County, Sichuan Province | -- | 2007.12.25 | Muscle | √ |
| SCGB00065 | Lady Amherst’s Pheasant | *Chrysolophus amherstiae* | Muping Town, Baoxing County, Sichuan Province | -- | 2007.12.25 | Muscle | × |
| SCGB00066 | Lady Amherst’s Pheasant | *Chrysolophus amherstiae* | Muping Town, Baoxing County, Sichuan Province | -- | 2007.12.25 | Muscle | × |
| SCGB00068 | Lady Amherst’s Pheasant | *Chrysolophus amherstiae* | Muping Town, Baoxing County, Sichuan Province | -- | 2007.12.25 | Muscle | × |
| SCGB00069 | Lady Amherst’s Pheasant | *Chrysolophus amherstiae* | Muping Town, Baoxing County, Sichuan Province | -- | 2007.12.25 | Muscle | √ |
| SCGB00070 | Lady Amherst’s Pheasant | *Chrysolophus amherstiae* | Muping Town, Baoxing County, Sichuan Province | -- | 2007.12.25 | Muscle | × |
| SCGB00071 | Lady Amherst’s Pheasant | *Chrysolophus amherstiae* | Muping Town, Baoxing County, Sichuan Province | -- | 2007.12.25 | Muscle | × |
| SCGB00179 | Lady Amherst’s Pheasant | *Chrysolophus amherstiae* | Pixian County, Sichuan Province | ♂ | 2007.9.28 | Blood | √ |
| SCGB00180 | Lady Amherst’s Pheasant | *Chrysolophus amherstiae* | Pixian County, Sichuan Province | ♂ | 2007.9.28 | Blood | × |
| SCGB00042 | Lady Amherst’s Pheasant | *Chrysolophus amherstiae* | Pixian County, Sichuan Province | ♂ | 2011.9.26 | whole | √ |
| SCGB00217 | Lady Amherst’s Pheasant | *Chrysolophus amherstiae* | Pixian County, Sichuan Province | -- | 2011.9.26 | Muscle | × |
| SCGB00038 | White Eared Pheasant | *Crossoptilon crossoptilon* | Xichang Zoo, Liangshan Prefecture, Sichuan Province | -- | 2018.3.26 | Muscle | √ |
| SCGB00041 | White Eared Pheasant | *Crossoptilon crossoptilon* | Xichang Zoo, Liangshan Prefecture, Sichuan Province | ♀ | 2018.3.26 | whole | √ |
| SCGB00134 | White Eared Pheasant | *Crossoptilon crossoptilon* | Pamuling, Yajiang County, Sichuan Province | -- | 2007.6.29 | Blood | × |
| SCGB00135 | White Eared Pheasant | *Crossoptilon crossoptilon* | Pamuling, Yajiang County, Sichuan Province | ♂ | 2007.6.29 | liver | √ |
| SCGB00191 | White Eared Pheasant | *Crossoptilon crossoptilon* | Pamuling, Yajiang County, Sichuan Province | ♂ | 2007.9.28 | Blood | × |
| SCGB00192 | White Eared Pheasant | *Crossoptilon crossoptilon* | Pamuling, Yajiang County, Sichuan Province | ♂ | 2007.9.28 | Blood | √ |
| SCGB00019 | White Eared Pheasant | *Crossoptilon crossoptilon* | Pamuling, Yajiang County, Sichuan Province | -- | 2007.9.28 | Muscle | × |
| SCGB00020 | White Eared Pheasant | *Crossoptilon crossoptilon* | Pamuling, Yajiang County, Sichuan Province | -- | 2007.9.28 | Muscle | × |
| SCGB00202 | White Eared Pheasant | *Crossoptilon crossoptilon* | Pamuling, Yajiang County, Sichuan Province | -- | 2007.11.7 | Blood | × |
| SCGB00185 | Reeves’s Pheasant | *Syrmaticus reevesii* | Pixian County, Sichuan Province | ♂ | 2007.9.28 | Blood | √ |
| SCGB00186 | Reeves’s Pheasant | *Syrmaticus reevesii* | Pixian County, Sichuan Province | ♂ | 2007.9.28 | Blood | × |
| SCGB00195 | Brown Eared-pheasant | *Crossoptilon mantchuricum* | Machang Village, Huanglong County, Yan'an City, Shaanxi Province | -- | 2010.8.1 | liver | √ |
| SCGB00196 | Brown Eared Pheasant | *Crossoptilon mantchuricum* | Machang Village, Huanglong County, Yan'an City, Shaanxi Province | -- | 2010.8.1 | Muscle | √ |
| SCGB00197 | Brown Eared Pheasant | *Crossoptilon mantchuricum* | Machang Village, Huanglong County, Yan'an City, Shaanxi Province | -- | 2010.8.1 | liver | × |
| SCGB00198 | Brown Eared Pheasant | *Crossoptilon mantchuricum* | Machang Village, Huanglong County, Yan'an City, Shaanxi Province | -- | 2010.8.1 | heart | × |
| SCGB00201 | Temminck’s Tragopan | *Tragopan temminckii* | Sichuan Tangjiahe National Nature Reserve | ♂ | 2008.9.20 | Muscle | √ |
| SCGB00215 | Temminck’s Tragopan | *Tragopan temminckii* | Pixian County, Sichuan Province | -- | 2011.9.26 | Muscle | √ |
| SCGB00181 | Golden Pheasant | *Chrysolophus pictus* | Pixian County, Sichuan Province | ♂ | 2007.9.28 | Blood | √ |
| SCGB00182 | Golden Pheasant | *Chrysolophus pictus* | Pixian County, Sichuan Province | ♀ | 2007.9.28 | Blood | × |
| SCGB00203 | Golden Pheasant | *Chrysolophus pictus* | Pixian County, Sichuan Province | ♂ | 2007.9.28 | Muscle | √ |
| SCGB00183 | Blue Eared Pheasant | *Crossoptilon auritum* | Chengdu Zoo, Sichuan Province | -- | 2005.11.11 | Blood | √ |
| SCGB00187 | Blue Eared Pheasant | *Crossoptilon auritum* | Pixian County, Sichuan Province | ♂ | 2007.9.28 | Blood | × |
| SCGB00188 | Blue Eared Pheasant | *Crossoptilon auritum* | Pixian County, Sichuan Province | ♀ | 2007.9.28 | Blood | √ |
| SCGB00034 | Chinese Monal | *Lophophorus lhuysii* | Pixian County, Sichuan Province | ♂ | 2011.10.11 | whole | √ |
| SCGB00100 | Chinese Monal | *Lophophorus lhuysii* | Pixian County, Sichuan Province | -- | 2010.3.29 | Muscle | √ |
| SCGB00075 | Chinese Monal | *Lophophorus lhuysii* | Pixian County, Sichuan Province | -- | 2010.3.29 | Muscle | √ |
| SCGB00200 | Chinese Monal | *Lophophorus lhuysii* | Pixian County, Sichuan Province | -- | 2008.11.21 | Blood | √ |
| SCGB00212 | Koklass Pheasant | *Pucrasia macrolopha* | Pixian County, Sichuan Province | -- | 2011.9.26 | Muscle | √ |
| SCGB00282 | Koklass Pheasant | *Pucrasia macrolopha* | Pamuling, Yajiang County, Sichuan Province | ♂ | 2007.11.7 | whole | √ |
| SCGB00287 | Sichuan Hill Partridge | *Arborophila rufipectus* | Sichuan Laojunshan National Nature Reserve | ♀ | 2012.4.29 | whole | × |
| SCGB00288 | Sichuan Hill Partridge | *Arborophila rufipectus* | Sichuan Laojunshan National Nature Reserve | ♂ | 2012.4.29 | whole | × |
| SCGB00289 | Sichuan Hill Partridge | *Arborophila rufipectus* | Sichuan Laojunshan National Nature Reserve | ♂ | 2012.4.29 | whole | × |
| SCGB00290 | Sichuan Hill Partridge | *Arborophila rufipectus* | Sichuan Laojunshan National Nature Reserve | ♂ | 2012.4.30 | whole | × |
| SCGB00291 | Sichuan Hill Partridge | *Arborophila rufipectus* | Sichuan Laojunshan National Nature Reserve | -- | 2012.7.7 | Muscle | × |
| SCGB00292 | Sichuan Hill Partridge | *Arborophila rufipectus* | Sichuan Laojunshan National Nature Reserve | -- | 2012.6.30 | Muscle | × |
| SCGB00293 | Sichuan Hill Partridge | *Arborophila rufipectus* | Sichuan Laojunshan National Nature Reserve | -- | 2012.7.7 | Muscle | × |
| SCGB00294 | Sichuan Hill Partridge | *Arborophila rufipectus* | Sichuan Laojunshan National Nature Reserve | -- | 2012.7.7 | Muscle | × |
| SCGB00295 | Sichuan Hill Partridge | *Arborophila rufipectus* | Sichuan Laojunshan National Nature Reserve | ♀ | 2012.5.24 | whole | √ |
| SCGB00296 | Sichuan Hill Partridge | *Arborophila rufipectus* | Sichuan Laojunshan National Nature Reserve | ♀ | 2012.5.24 | whole | √ |
| SCGB00297 | Sichuan Hill Partridge | *Arborophila rufipectus* | Sichuan Laojunshan National Nature Reserve | ♂ | 2012.5 .24 | whole | √ |
| SCGB00299 | Sichuan Hill Partridge | *Arborophila rufipectus* | Sichuan Laojunshan National Nature Reserve | -- | 2012.5.30 | liver | √ |
| SCGB00300 | Sichuan Hill Partridge | *Arborophila rufipectus* | Sichuan Laojunshan National Nature Reserve | -- | 2012.5.30 | Muscle | √ |
| SCGB00304 | Sichuan Hill Partridge | *Arborophila rufipectus* | Sichuan Laojunshan National Nature Reserve | ♀ | 2012.7.11 | Blood | √ |
| SCGB00305 | Sichuan Hill Partridge | *Arborophila rufipectus* | Sichuan Laojunshan National Nature Reserve | ♂ | 2012.6.3 | Blood | × |
| SCGB00306 | Sichuan Hill Partridge | *Arborophila rufipectus* | Sichuan Laojunshan National Nature Reserve | ♀ | 2012.6.23 | Blood | √ |
| SCGB00307 | Sichuan Hill Partridge | *Arborophila rufipectus* | Sichuan Laojunshan National Nature Reserve | ♀ | 2012.6.30 | Blood | √ |
| SCGB00308 | Sichuan Hill Partridge | *Arborophila rufipectus* | Sichuan Laojunshan National Nature Reserve | ♂ | 2012.7.24 | Blood | √ |
| SCGB00309 | Sichuan Hill Partridge | *Arborophila rufipectus* | Sichuan Laojunshan National Nature Reserve | ♂ | 2012.8.26 | Blood | √ |
| SCGB00310 | Sichuan Hill Partridge | *Arborophila rufipectus* | Sichuan Laojunshan National Nature Reserve | ♀ | 2012.9.5 | Blood | × |
| SCGB00311 | Sichuan Hill Partridge | *Arborophila rufipectus* | Sichuan Laojunshan National Nature Reserve | ♀ | 2012.7.28 | Blood | √ |
| SCGB00312 | Sichuan Hill Partridge | *Arborophila rufipectus* | Sichuan Laojunshan National Nature Reserve | ♀ | 2012.4.21 | Blood | √ |
| SCGB00313 | Sichuan Hill Partridge | *Arborophila rufipectus* | Sichuan Laojunshan National Nature Reserve | ♀ | 2012.6.20 | Blood | √ |
| SCGB00314 | Sichuan Hill Partridge | *Arborophila rufipectus* | Sichuan Laojunshan National Nature Reserve | ♀ | 2012.7.18 | Blood | √ |
| SCGB00315 | Sichuan Hill Partridge | *Arborophila rufipectus* | Sichuan Laojunshan National Nature Reserve | ♀ | 2012.8.20 | Blood | √ |
| SCGB00316 | Sichuan Hill Partridge | *Arborophila rufipectus* | Sichuan Laojunshan National Nature Reserve | ♂ | 2012.8.6 | Blood | √ |
| SCGB00317 | Sichuan Hill Partridge | *Arborophila rufipectus* | Sichuan Laojunshan National Nature Reserve | ♂ | 2012.4.25 | Blood | √ |
| SCGB00318 | Sichuan Hill Partridge | *Arborophila rufipectus* | Sichuan Laojunshan National Nature Reserve | ♂ | 2012.4.25 | Blood | √ |
| SCGB00319 | Sichuan Hill Partridge | *Arborophila rufipectus* | Sichuan Laojunshan National Nature Reserve | ♀ | 2012.4.24 | Blood | √ |
| SCGB00320 | Sichuan Hill Partridge | *Arborophila rufipectus* | Sichuan Laojunshan National Nature Reserve | ♀ | 2012.4.24 | Blood | √ |
| SCGB00321 | Sichuan Hill Partridge | *Arborophila rufipectus* | Sichuan Laojunshan National Nature Reserve | ♂ | 2012.4.24 | Blood | √ |
| SCGB00136 | Buff-throated Partridge | *Tetraophasis szechenyii* | Pamuling, Yajiang County, Sichuan Province | ♂ | 2007.7.14 | Blood | × |
| SCGB00137 | Buff-throated Partridge | *Tetraophasis szechenyii* | Pamuling, Yajiang County, Sichuan Province | ♂ | 2007.7.14 | liver | √ |
| SCGB00138 | Buff-throated Partridge | *Tetraophasis szechenyii* | Pamuling, Yajiang County, Sichuan Province | ♂ | 2007.7.14 | Muscle | × |
| SCGB00139 | Buff-throated Partridge | *Tetraophasis szechenyii* | Pamuling, Yajiang County, Sichuan Province | ♂ | 2007.7.14 | heart | √ |
| SCGB00141 | Buff-throated Partridge | *Tetraophasis szechenyii* | Pamuling, Yajiang County, Sichuan Province | ♂ | 2007.9.25 | heart | × |
| SCGB00142 | Buff-throated Partridge | *Tetraophasis szechenyii* | Pamuling, Yajiang County, Sichuan Province | -- | 2007.9.25 | Blood | × |
| SCGB00143 | Buff-throated Partridge | *Tetraophasis szechenyii* | Pamuling, Yajiang County, Sichuan Province | -- | 2007.9.25 | Blood | × |
| SCGB00145 | Buff-throated Partridge | *Tetraophasis szechenyii* | Pamuling, Yajiang County, Sichuan Province | ♂ | 2007.9.25 | Blood | × |
| SCGB00149 | Buff-throated Partridge | *Tetraophasis szechenyii* | Pamuling, Yajiang County, Sichuan Province | ♂ | 2007.11.7 | Blood | × |
| SCGB00150 | Buff-throated Partridge | *Tetraophasis szechenyii* | Pamuling, Yajiang County, Sichuan Province | ♂ | 2007.11.7 | Blood | × |
| SCGB00151 | Buff-throated Partridge | *Tetraophasis szechenyii* | Pamuling, Yajiang County, Sichuan Province | ♂ | 2007.11.7 | Blood | √ |
| SCGB00152 | Buff-throated Partridge | *Tetraophasis szechenyii* | Pamuling, Yajiang County, Sichuan Province | ♀ | 2007.11.7 | Blood | √ |
| SCGB00153 | Buff-throated Partridge | *Tetraophasis szechenyii* | Pamuling, Yajiang County, Sichuan Province | ♂ | 2008.11.14 | Blood | × |
| SCGB00154 | Buff-throated Partridge | *Tetraophasis szechenyii* | Pamuling, Yajiang County, Sichuan Province | ♂ | 2008.11.14 | Blood | × |
| SCGB00155 | Buff-throated Partridge | *Tetraophasis szechenyii* | Pamuling, Yajiang County, Sichuan Province | ♂ | 2008.11.14 | Blood | × |
| SCGB00156 | Buff-throated Partridge | *Tetraophasis szechenyii* | Pamuling, Yajiang County, Sichuan Province | ♂ | 2008.11.14 | Blood | √ |
| SCGB00157 | Buff-throated Partridge | *Tetraophasis szechenyii* | Pamuling, Yajiang County, Sichuan Province | ♂ | 2008.11.15 | Blood | × |
| SCGB00158 | Buff-throated Partridge | *Tetraophasis szechenyii* | Pamuling, Yajiang County, Sichuan Province | ♂ | 2008.10.3 | Blood | × |
| SCGB00159 | Buff-throated Partridge | *Tetraophasis szechenyii* | Pamuling, Yajiang County, Sichuan Province | ♂ | 2008.1.3 | Blood | × |
| SCGB00160 | Buff-throated Partridge | *Tetraophasis szechenyii* | Pamuling, Yajiang County, Sichuan Province | ♂ | 2008.12.28 | Blood | × |
| SCGB00161 | Buff-throated Partridge | *Tetraophasis szechenyii* | Pamuling, Yajiang County, Sichuan Province | ♀ | 2008.12.28 | Blood | √ |
| SCGB00162 | Buff-throated Partridge | *Tetraophasis szechenyii* | Pamuling, Yajiang County, Sichuan Province | ♂ | 2008.11.16 | Blood | √ |
| SCGB00163 | Buff-throated Partridge | *Tetraophasis szechenyii* | Pamuling, Yajiang County, Sichuan Province | ♀ | 2008.11.16 | Blood | × |
| SCGB00164 | Buff-throated Partridge | *Tetraophasis szechenyii* | Pamuling, Yajiang County, Sichuan Province | ♀ | 2008.11.16 | Blood | √ |
| SCGB00165 | Buff-throated Partridge | *Tetraophasis szechenyii* | Pamuling, Yajiang County, Sichuan Province | ♀ | 2008.11.16 | Blood | × |
| SCGB00166 | Buff-throated Partridge | *Tetraophasis szechenyii* | Pamuling, Yajiang County, Sichuan Province | ♂ | 2007.9.25 | Blood | √ |
| SCGB00167 | Buff-throated Partridge | *Tetraophasis szechenyii* | Pamuling, Yajiang County, Sichuan Province | ♂ | 2007.9.25 | Muscle | × |
| SCGB00168 | Buff-throated Partridge | *Tetraophasis szechenyii* | Pamuling, Yajiang County, Sichuan Province | ♂ | 2007.9.25 | liver | × |
| SCGB00169 | Buff-throated Partridge | *Tetraophasis szechenyii* | Pamuling, Yajiang County, Sichuan Province | ♀ | 2007.9.25 | Blood | × |
| SCGB00170 | Buff-throated Partridge | *Tetraophasis szechenyii* | Pamuling, Yajiang County, Sichuan Province | ♂ | 2008.1.3 | Blood | × |
| SCGB00171 | Buff-throated Partridge | *Tetraophasis szechenyii* | Pamuling, Yajiang County, Sichuan Province | ♂ | 2008.1.3 | Blood | × |
| SCGB00172 | Buff-throated Partridge | *Tetraophasis szechenyii* | Pamuling, Yajiang County, Sichuan Province | ♀ | 2007.9.25 | Blood | × |
| SCGB00173 | Buff-throated Partridge | *Tetraophasis szechenyii* | Pamuling, Yajiang County, Sichuan Province | ♂ | 2008.12.29 | Blood | √ |
| SCGB00174 | Buff-throated Partridge | *Tetraophasis szechenyii* | Pamuling, Yajiang County, Sichuan Province | ♀ | 2008.11.16 | Blood | √ |
| SCGB00175 | Buff-throated Partridge | *Tetraophasis szechenyii* | Pamuling, Yajiang County, Sichuan Province | ♂ | 2008.11.16 | Blood | × |
| SCGB00176 | Buff-throated Partridge | *Tetraophasis szechenyii* | Pamuling, Yajiang County, Sichuan Province | ♀ | 2007.11.7 | Blood | √ |
| SCGB00177 | Buff-throated Partridge | *Tetraophasis szechenyii* | Pamuling, Yajiang County, Sichuan Province | ♀ | 2007.11.7 | Blood | √ |
| SCGB00218 | Buff-throated Partridge | *Tetraophasis szechenyii* | Pamuling, Yajiang County, Sichuan Province | ♂ | 2009.3.15 | Blood | × |
| SCGB00219 | Buff-throated Partridge | *Tetraophasis szechenyii* | Pamuling, Yajiang County, Sichuan Province | ♂ | 2099.3.15 | Blood | × |
| SCGB00220 | Buff-throated Partridge | *Tetraophasis szechenyii* | Pamuling, Yajiang County, Sichuan Province | ♂ | 2009.3.15 | Blood | × |
| SCGB00221 | Buff-throated Partridge | *Tetraophasis szechenyii* | Pamuling, Yajiang County, Sichuan Province | ♂ | 2009.3.15 | Blood | × |
| SCGB00222 | Buff-throated Partridge | *Tetraophasis szechenyii* | Pamuling, Yajiang County, Sichuan Province | ♂ | 2009.3.15 | Blood | × |
| SCGB00223 | Buff-throated Partridge | *Tetraophasis szechenyii* | Pamuling, Yajiang County, Sichuan Province | ♂ | 2009.3.15 | Blood | × |
| SCGB00224 | Buff-throated Partridge | *Tetraophasis szechenyii* | Pamuling, Yajiang County, Sichuan Province | ♂ | 2009.3.15 | Blood | × |
| SCGB00225 | Buff-throated Partridge | *Tetraophasis szechenyii* | Pamuling, Yajiang County, Sichuan Province | ♀ | 2009.3.15 | Blood | × |
| SCGB00226 | Buff-throated Partridge | *Tetraophasis szechenyii* | Pamuling, Yajiang County, Sichuan Province | ♂ | 2009.3.15 | Blood | × |
| SCGB00227 | Buff-throated Partridge | *Tetraophasis szechenyii* | Pamuling, Yajiang County, Sichuan Province | ♂ | 2009.3.15 | Blood | × |
| SCGB00228 | Buff-throated Partridge | *Tetraophasis szechenyii* | Pamuling, Yajiang County, Sichuan Province | -- | 2009.3.15 | Blood | × |
| SCGB00229 | Buff-throated Partridge | *Tetraophasis szechenyii* | Pamuling, Yajiang County, Sichuan Province | -- | 2009.3.15 | Blood | × |
| SCGB00230 | Buff-throated Partridge | *Tetraophasis szechenyii* | Pamuling, Yajiang County, Sichuan Province | -- | 2009.3.15 | Blood | × |
| SCGB00231 | Buff-throated Partridge | *Tetraophasis szechenyii* | Pamuling, Yajiang County, Sichuan Province | -- | 2009.3.15 | Blood | × |
| SCGB00232 | Buff-throated Partridge | *Tetraophasis szechenyii* | Pamuling, Yajiang County, Sichuan Province | -- | 2009.3.15 | Blood | × |
| SCGB00233 | Buff-throated Partridge | *Tetraophasis szechenyii* | Pamuling, Yajiang County, Sichuan Province | -- | 2009.3.15 | Blood | × |
| SCGB00234 | Buff-throated Partridge | *Tetraophasis szechenyii* | Pamuling, Yajiang County, Sichuan Province | -- | 2009.3.15 | Blood | × |
| SCGB00235 | Buff-throated Partridge | *Tetraophasis szechenyii* | Pamuling, Yajiang County, Sichuan Province | -- | 2009.3.15 | Blood | × |
| SCGB00236 | Buff-throated Partridge | *Tetraophasis szechenyii* | Pamuling, Yajiang County, Sichuan Province | -- | 2009.3.15 | Blood | × |
| SCGB00237 | Buff-throated Partridge | *Tetraophasis szechenyii* | Pamuling, Yajiang County, Sichuan Province | -- | 2009.3.15 | Blood | × |
| SCGB00238 | Buff-throated Partridge | *Tetraophasis szechenyii* | Pamuling, Yajiang County, Sichuan Province | -- | 2009.3.15 | Blood | × |
| SCGB00239 | Buff-throated Partridge | *Tetraophasis szechenyii* | Pamuling, Yajiang County, Sichuan Province | -- | 2009.3.15 | Blood | × |
| SCGB00262 | Buff-throated Partridge | *Tetraophasis szechenyii* | Pamuling, Yajiang County, Sichuan Province | ♂ | 2009.3.15 | Blood | × |
| SCGB00263 | Buff-throated Partridge | *Tetraophasis szechenyii* | Pamuling, Yajiang County, Sichuan Province | ♂ | 2009.3.15 | Blood | × |
| SCGB00264 | Buff-throated Partridge | *Tetraophasis szechenyii* | Pamuling, Yajiang County, Sichuan Province | ♀ | 2009.3.15 | Blood | × |
| SCGB00265 | Buff-throated Partridge | *Tetraophasis szechenyii* | Pamuling, Yajiang County, Sichuan Province | ♀ | 2009.3.15 | Blood | × |
| SCGB00266 | Buff-throated Partridge | *Tetraophasis szechenyii* | Pamuling, Yajiang County, Sichuan Province | ♀ | 2009.3.15 | Blood | × |
| SCGB00267 | Buff-throated Partridge | *Tetraophasis szechenyii* | Pamuling, Yajiang County, Sichuan Province | ♀ | 2009.3.15 | Blood | × |
| SCGB00268 | Buff-throated Partridge | *Tetraophasis szechenyii* | Pamuling, Yajiang County, Sichuan Province | ♀ | 2009.3.15 | Blood | × |
| SCGB00269 | Buff-throated Partridge | *Tetraophasis szechenyii* | Pamuling, Yajiang County, Sichuan Province | ♀ | 2009.3.15 | Blood | × |
| SCGB00270 | Buff-throated Partridge | *Tetraophasis szechenyii* | Pamuling, Yajiang County, Sichuan Province | ♂ | 2009.3.16 | Blood | × |
| SCGB00271 | Buff-throated Partridge | *Tetraophasis szechenyii* | Pamuling, Yajiang County, Sichuan Province | ♂ | 2009.3.16 | Blood | × |
| SCGB00272 | Buff-throated Partridge | *Tetraophasis szechenyii* | Pamuling, Yajiang County, Sichuan Province | ♂ | 2009.4.9 | Blood | × |
| SCGB00273 | Buff-throated Partridge | *Tetraophasis szechenyii* | Pamuling, Yajiang County, Sichuan Province | ♀ | 2009.4.9 | Blood | × |
| SCGB00274 | Buff-throated Partridge | *Tetraophasis szechenyii* | Pamuling, Yajiang County, Sichuan Province | ♀ | 2009.4.9 | Blood | × |
| SCGB00275 | Buff-throated Partridge | *Tetraophasis szechenyii* | Pamuling, Yajiang County, Sichuan Province | ♀ | 2009.3.15 | Blood | × |
| SCGB00276 | Buff-throated Partridge | *Tetraophasis szechenyii* | Pamuling, Yajiang County, Sichuan Province | ♀ | 2009.3.15 | Blood | × |
| SCGB00277 | Buff-throated Partridge | *Tetraophasis szechenyii* | Pamuling, Yajiang County, Sichuan Province | ♂ | 2009.3.15 | Blood | × |
| SCGB00280 | Buff-throated Partridge | *Tetraophasis szechenyii* | Pamuling, Yajiang County, Sichuan Province | ♂ | 2007.11.14 | Muscle | √ |
| SCGB00281 | Buff-throated Partridge | *Tetraophasis szechenyii* | Pamuling, Yajiang County, Sichuan Province | -- | 2009.6.23 | Muscle | × |
| SCGB00421 | Common Quail | *Coturnix coturnix* | Guangyuan City, Sichuan Province | ♀ | 2018.6.24 | Muscle | √ |
| SCGB00033 | Common Pheasant | *Phasianus colchicus* | Nanchong City, Sichuan Province | -- | 2014.12.16 | Muscle | × |
| SCGB00056 | Common Pheasant | *Phasianus colchicus* | Nanchong City, Sichuan Province | -- | 2014.12.16 | Muscle | × |
| SCGB00057 | Common Pheasant | *Phasianus colchicus* | Nanchong City, Sichuan Province | -- | 2014.12.16 | Muscle | × |
| SCGB00067 | Common Pheasant | *Phasianus colchicus* | Nanchong City, Sichuan Province | -- | 2014.12.16 | Muscle | × |
| SCGB00184 | Common Pheasant | *Phasianus colchicus* | Pixian County, Sichuan Province | ♀ | 2007.9.28 | Blood | × |
| SCGB00205 | Common Pheasant | *Phasianus colchicus* | Tibet | ♂ | 2008.2.25 | Muscle | √ |

Note: “√” : obvious bands were detected in DNA extraction; “×” : no obvious band was detected in DNA extraction; “--” : gender unknown

**Table S2a** PCR reaction system

| Components | 20 ul system |
| --- | --- |
| 1.1×T3 Super PCR Mix | 17 ul |
| 10 uM Forward Primer | 1 ul |
| 10 uM Reverse Primer | 1 ul |
| Stencil DNA | 1 ul |

Note: 1.1×T3 Super PCR Mix was procured from Chengdu Tsingke Biotech Company, and the Stencil DNA was DNA of various pheasant species with qualified quality assessment.

**Table S2b** PCR reaction system

| Components | 20 ul system |
| --- | --- |
| 1.1×T3 Super PCR Mix | 15~17 ul |
| 10 uM Forward Primer | 1 ul |
| 10 uM Reverse Primer | 1 ul |
| Stencil DNA | 1~3 ul |

Note: 1.1×T3 Super PCR Mix was procured from Chengdu Tsingke Biotech Company, and the Stencil DNA was DNA of various pheasant species with qualified quality assessment.

**Table S2c** PCR reaction system

| Components | 20 ul system |
| --- | --- |
| 1.1×T3 Super PCR Mix | 17 ul |
| 10 uM Fluorescent Forward Primer | 1 ul |
| 10 uM Fluorescent Reverse Primer | 1 ul |
| Stencil DNA | 1 ul |

Note: 1.1×T3 Super PCR Mix was purchased from Chengdu Tsingke Biotech Company, and the Stencil was DNA of Sichuan Hill Partridge, Blood Pheasant and Buff-throated Partridge with qualified quality assessment.

**Table S3a** PCR reaction procedures

| Procedures | Temperature | Time | Number of cycles |
| --- | --- | --- | --- |
| Pre-mutability | 98 °C | 2 minutes | 1 |
| Mutability | 98 °C | 10 seconds | 35 |
| Annealing | 52 °C、55 °C、60 °C、65 °C | 30 seconds |  |
| Extension | 72 °C | 10 seconds |  |
| Ultimate Extension | 72 °C | 2 minutes | 1 |
| Conservation | 4 °C | ∞ |  |

Note: 4 temperature gradients of 52 °C, 55 °C, 60 °C and 65 °C were set for the ideal annealing temperature.

**Table S3b** PCR reaction procedures

| Procedures | Temperature | Time | Number of cycles |
| --- | --- | --- | --- |
| Pre-mutability | 98 °C | 2 minutes | 1 |
| Mutability | 98 °C | 10 seconds | 35 |
| Annealing | 60 °C | 30 seconds |  |
| Extension | 72 °C | 10 seconds |  |
| Ultimate Extension | 72 °C | 2 minutes | 1 |
| Conservation | 4 °C | ∞ |  |

**Figure S1** Number of genes with common microsatellite loci in exonic regions

**Table S4** Information of 49 potential universal microsatellite loci

| Name of microsatellite loci | Core Sequence | Sequence of PCR primers |
| --- | --- | --- |
| pha1 | GAGC | F:CAACCCCGACGCTATCTCAG |
|  |  | R:TTGCCGGTGTCACCACAATA |
| pha2 | TTTC | F:CCTGCTTTGCCCTTTCCCTA |
|  |  | R:GGCAAAGTTACGCCGTTGTT |
| pha9 | CTTC | F:ACCTGGGATTTGGGCCATTT |
|  |  | R:GTGGCTAAATGCTGGCAATCA |
| pha13 | ATTT | F:GCTGGTCTGGCTGCAATAGA |
|  |  | R:ACAGAAAGTCTCCTGCTTTGA |
| pha14 | AAAT | F:ACACTGCCATCCATTGCTGT |
|  |  | R:ACGCTTCCTGGAGGGGAATA |
| pha15 | AAAT | F:GGCTGTGAGCAAGAGATGGA |
|  |  | R:TCCCAGGACATTTGTTGTTCAC |
| pha16 | TTTA | F:ACTCTGCAAGAAGCCCCAAA |
|  |  | R:AGGCTTCCTGCTCAAGCTTT |
| pha18 | GATG | F:ACCCTTTGCACTCTAACGCT |
|  |  | R:CCAGGGGAAATGGTGGTGAA |
| pha19 | TTTA | F:CGGAGCATTAAGTGGCAGAT |
|  |  | R:CACCGCCGGTTGTTCTTCTA |
| pha30 | TTG | F:GAAGTTTGAAAGCTCGGCCTT |
|  |  | R:GCCAGTGCACACATCAACAG |
| pha31 | ATT | F:TCTGACACAGACCGAGTACCT |
|  |  | R:CACCCCTGAAAGCGTGTTTG |
| pha32 | CAT | F:GGAGGAGGGATCCCATTTCC |
|  |  | R:AGGTTTCACATCTGCCAGCT |
| pha35 | CT | F:TTGTTGTGAGGCGAGCGATA |
|  |  | R:CGGACTCTGCACTGCATGAT |
| pha43 | AT | F:ATGCTATGCACCTCGGGTTG |
|  |  | R:AGGCAACCTGCTTTTCAGAGA |
| pha49 | GT | F:AACCCAACCATCTCCCTGTG |
|  |  | R:TGGCAAGGAAGGAGGAATCG |
| pha50 | TG | F:TCTGTGTGGAAGGCCTGAAC |
|  |  | R:CAAGCACCAATCAGAAACCACA |
| pha51 | AG | F:GCTGCTGACCCCAAGTGTTT |
|  |  | R:TCCATCTTTTGAGGTAGCTGA |
| pha53 | CT | F:GCATGTTTCTTAGAGTGAGAGCA |
|  |  | R:AGACGATCGGAAAGGAATGTGA |
| pha55 | AT | F:AGAAGCAGGCATAGCACTACA |
|  |  | R:AACTGCTGTGTTCCTGTCCA |
| pha57 | GA | F:ACCGTGAACTCCTGTTGCAT |
|  |  | R:TCCAACACCATCTCGTACGG |
| pha58 | GT | F:CTGTCCTCCCTGCTCCATTG |
|  |  | R:CTCCCCGTGTAATTGTGCCT |
| pha60 | GA | F:CCTCTGCCTGCCTGCTAATT |
|  |  | R:TGACTGTGAACCTCGACAGC |
| pha62 | GA | F:CTGGACTCCTCGGATGAACG |
|  |  | R:CTTGGTGTTGTTATGGCGAGT |
| pha64 | AG | F:CTTCCTACTACCAGCAAGGCA |
|  |  | R:TCGTGCATTTTCCCCTGGAA |
| pha69 | GA | F:TCATTTGAGCTGTCCACCGT |
|  |  | R:GTCCATGCCATTGTCTCCGT |
| pha70 | GA | F:GAGTCCGGATGCGGTTAGAG |
|  |  | R:TCTGAGGCCGTTTGTGTCTC |
| pha71 | AT | F:TCACGAAACAGGAAAACGAGT |
|  |  | R:TTCACAGGGTGGGAACTTGG |
| pha72 | CT | F:GGGGACTGGACTTTTCGAATCA |
|  |  | R:AGCATTTCAGGGAGAGAGCAC |
| pha74 | CA | F:GGGAGAGGAGAAGAGACCGA |
|  |  | R:CCCCTTACAGCCTGGCAATA |
| pha77 | GA | F:TCTGGATGGCCCTTTCACAA |
|  |  | R:CCTTCTGTCCCTGTCGCATT |
| pha79 | TA | F:CACCCTGGCTGGAGATCAAG |
|  |  | R:GCTGCTTTGATGCAAATGCC |
| pha80 | AG | F:TCAGACCAGCCAGCGAAAAT |
|  |  | R:ACCTTGAGGCTTTCAGCTGA |
| pha85 | CT | F:GGACTCCCCAAAACCACACT |
|  |  | R:TGCTGGCTCTTTAAGACCGA |
| pha87 | AG | F:CGGGCACTTCTGTCAAAACG |
|  |  | R:TCCGAGCTGGAGTTGCATTT |
| phaA1 | AAC | F:TGCTCTGCTCAGAAGACAGAC |
|  |  | R:GGTGCAGGTACCTCAGAACC |
| phaA2 | AGG | F:CCCTTTCCTGGGTGAAGGAC |
|  |  | R:AATACCTCCGCCTGCCTTTC |
| phaA3 | CCT | F:GCCTAAACGCACTTGGTTGG |
|  |  | R:CTTCGTGCCATCAGCCAGA |
| phaA4 | TAT | F:GTTGGAAGGCTTTGCAGCTC |
|  |  | R:TCTCCAAGCAAGCAGTAGGC |
| phaA6 | AAT | F:GCTGTAAAGAGGAGTCGCAGT |
|  |  | R:GCCAAGCTTGTGACAGGCTA |
| phaA7 | AAT | F:GGCTGAAAACTGGGAAACGT |
|  |  | R:GCCCGCACTACCATACATCA |
| phaG1 | ATA | F:CACACAGCTTCAGGGAGTCA |
|  |  | R:GGCTTTTGATTGTCTCACGCT |
| phaG4 | TGC | F:AGGTGTGCTCCGTAGAGACT |
|  |  | R:CCTGCGAAGAGTCCTCATGG |
| phaG5 | GCA | F:ACTGTTTCAGCTGCGCAAAC |
|  |  | R:TCTGACGAGATGGGCGTAGA |
| phaG8 | CAA | F:ACTCTGCTATCCTACTCCAGCA |
|  |  | R:CAGCCACCAGCCTAGGTTTT |
| phaJ5 | AAT | F:CAAACGTGGGTTGCAGTAGT |
|  |  | R:TCCATTCGAAAGCAGTGGGT |
| phaJ6 | TCC | F:GATCGCTGCTCCTCCTCTTC |
|  |  | R:TCTGACAGCATGGACGACAC |
| phaJ8 | TAA | F:GCGGTTGCCTGTCAGTAGTT |
|  |  | R:TTAACCACACGGGGTGTTCC |
| phaJ9 | TTC | F:GCTGGGCTTGATCTTCAGGA |
|  |  | R:TCACAGCCCAACGTTCTGAT |
| phaK2 | ACC | F:AGGATTCCTGGCTTGAGCTA |
|  |  | R:TGCTGGGAGCCTGTTTTCTT |

**Table S5a** Genetic diversity analysis of the Sichuan Hill Partridge population in Laojunshan based on microsatellite markers

| Locus | K | N | Ho | He | PIC | P-value |
| --- | --- | --- | --- | --- | --- | --- |
| pha1 | 3 | 21 | 0.286 | 0.257 | 0.229 | 1.0000 |
| pha2 | 2 | 21 | 0.048 | 0.136 | 0.124 | 0.0701 |
| pha9 | 3 | 21 | 0.190 | 0.390 | 0.336 | 0.0053 |
| pha14 | 2 | 21 | 0.000 | 0.093 | 0.087 | 0.0252 |
| pha18 | 2 | 21 | 0.000 | 0.177 | 0.157 | 0.0017 |
| pha35 | 4 | 21 | 0.619 | 0.570 | 0.492 | 0.1536 |
| pha49 | 3 | 21 | 0.952 | 0.598 | 0.503 | 0.0003 |
| pha50 | 3 | 21 | 0.333 | 0.361 | 0.315 | 0.0605 |
| pha51 | 3 | 21 | 0.429 | 0.480 | 0.415 | 0.0015 |
| pha53 | 2 | 21 | 0.571 | 0.418 | 0.325 | 0.1325 |
| pha55 | 3 | 21 | 0.952 | 0.577 | 0.466 | 0.0000 |
| pha57 | 3 | 21 | 0.810 | 0.528 | 0.423 | 0.0108 |
| pha58 | 2 | 21 | 0.000 | 0.093 | 0.087 | 0.0242 |
| pha62 | 4 | 21 | 0.190 | 0.224 | 0.211 | 0.1408 |
| pha69 | 2 | 21 | 0.857 | 0.502 | 0.370 | 0.0015 |
| pha71 | 3 | 21 | 0.905 | 0.596 | 0.490 | 0.0000 |
| pha77 | 3 | 21 | 0.571 | 0.431 | 0.350 | 0.3247 |
| pha79 | 4 | 21 | 0.905 | 0.666 | 0.585 | 0.0000 |
| pha80 | 2 | 21 | 0.095 | 0.093 | 0.087 | 1.0000 |
| pha85 | 5 | 21 | 1.000 | 0.670 | 0.594 | 0.0000 |
| pha87 | 3 | 21 | 0.571 | 0.539 | 0.455 | 0.0007 |
| pha30 | 2 | 21 | 0.238 | 0.215 | 0.188 | 1.0000 |
| phaA1 | 3 | 21 | 0.429 | 0.591 | 0.488 | 0.0068 |
| phaA3 | 2 | 21 | 0.238 | 0.345 | 0.280 | 0.1942 |
| phaA4 | 2 | 21 | 0.048 | 0.136 | 0.124 | 0.0747 |
| phaA6 | 4 | 21 | 0.619 | 0.544 | 0.464 | 0.0142 |
| phaA7 | 2 | 21 | 0.524 | 0.396 | 0.312 | 0.2641 |
| phaG1 | 3 | 21 | 0.190 | 0.180 | 0.166 | 1.0000 |
| phaG4 | 3 | 21 | 0.095 | 0.138 | 0.130 | 0.0699 |
| phaG5 | 2 | 21 | 0.048 | 0.136 | 0.124 | 0.0734 |
| phaJ5 | 4 | 21 | 0.667 | 0.582 | 0.497 | 0.0006 |
| phaJ6 | 2 | 21 | 0.476 | 0.372 | 0.297 | 0.2877 |
| phaJ8 | 2 | 21 | 0.857 | 0.502 | 0.370 | 0.0012 |
| Average | 2.7879 | - | 0.4458 | 0.3799 | 0.3194 | - |

**Table S5b** Genetic diversity analysis of the Blood Pheasant population in Pamuling based on microsatellite markers

| Locus | K | N | Ho | He | PIC | P-value |
| --- | --- | --- | --- | --- | --- | --- |
| pha1 | 3 | 19 | 0.842 | 0.599 | 0.496 | 0.0197 |
| pha16 | 2 | 19 | 0.526 | 0.398 | 0.313 | 0.2607 |
| pha49 | 3 | 19 | 0.158 | 0.152 | 0.142 | 1.0000 |
| pha50 | 7 | 19 | 0.895 | 0.679 | 0.613 | 0.0308 |
| pha51 | 5 | 19 | 1.000 | 0.706 | 0.636 | 0.0075 |
| pha53 | 2 | 19 | 0.789 | 0.491 | 0.364 | 0.0111 |
| pha55 | 2 | 19 | 0.895 | 0.508 | 0.372 | 0.0009 |
| pha57 | 3 | 19 | 0.947 | 0.563 | 0.445 | 0.0003 |
| pha60 | 5 | 19 | 1.000 | 0.673 | 0.592 | 0.0000 |
| pha64 | 3 | 19 | 0.421 | 0.364 | 0.327 | 1.0000 |
| pha69 | 2 | 19 | 0.421 | 0.444 | 0.339 | 1.0000 |
| pha70 | 2 | 19 | 0.421 | 0.341 | 0.277 | 0.5363 |
| pha77 | 3 | 19 | 1.000 | 0.582 | 0.473 | 0.0000 |
| pha79 | 3 | 19 | 0.947 | 0.622 | 0.534 | 0.0015 |
| pha80 | 2 | 19 | 0.526 | 0.398 | 0.313 | 0.2591 |
| pha85 | 2 | 19 | 0.789 | 0.491 | 0.364 | 0.0128 |
| pha87 | 2 | 19 | 0.632 | 0.444 | 0.339 | 0.1078 |
| phaA1 | 2 | 19 | 0.158 | 0.149 | 0.135 | 1.0000 |
| phaA3 | 3 | 19 | 0.158 | 0.240 | 0.216 | 0.2538 |
| phaA7 | 2 | 19 | 0.737 | 0.478 | 0.357 | 0.0398 |
| phaG1 | 2 | 19 | 0.211 | 0.193 | 0.171 | 1.0000 |
| phaG5 | 2 | 19 | 0.526 | 0.398 | 0.313 | 0.2659 |
| phaJ8 | 2 | 19 | 0.947 | 0.512 | 0.374 | 0.0001 |
| phaK2 | 2 | 19 | 0.211 | 0.193 | 0.171 | 1.0000 |
| Average | 2.7500 | - | 0.6315 | 0.4424 | 0.3615 | - |

**Table S5c** Genetic diversity analysis of the Buff-throated Partridge population in Pamuling based on microsatellite markers

| Locus | K | N | Ho | He | PIC | P-value |
| --- | --- | --- | --- | --- | --- | --- |
| pha9 | 2 | 14 | 0.357 | 0.495 | 0.363 | 0.5709 |
| pha14 | 2 | 14 | 0.286 | 0.254 | 0.215 | 1.0000 |
| pha35 | 4 | 14 | 1.000 | 0.733 | 0.653 | 0.0021 |
| pha43 | 3 | 14 | 0.929 | 0.553 | 0.424 | 0.0041 |
| pha51 | 2 | 14 | 0.357 | 0.304 | 0.250 | 1.0000 |
| pha53 | 3 | 14 | 0.929 | 0.653 | 0.552 | 0.1237 |
| pha55 | 2 | 14 | 0.714 | 0.476 | 0.354 | 0.0909 |
| pha60 | 3 | 14 | 1.000 | 0.646 | 0.551 | 0.0015 |
| pha62 | 2 | 14 | 0.357 | 0.389 | 0.305 | 1.0000 |
| pha74 | 2 | 14 | 0.429 | 0.476 | 0.354 | 1.0000 |
| pha71 | 3 | 14 | 1.000 | 0.582 | 0.465 | 0.0007 |
| pha77 | 2 | 14 | 0.929 | 0.516 | 0.374 | 0.0054 |
| pha79 | 3 | 14 | 0.929 | 0.627 | 0.537 | 0.0229 |
| pha80 | 2 | 14 | 0.857 | 0.508 | 0.370 | 0.0223 |
| pha72 | 2 | 14 | 0.429 | 0.349 | 0.280 | 1.0000 |
| phaA1 | 2 | 14 | 0.500 | 0.389 | 0.305 | 0.5129 |
| phaA6 | 2 | 14 | 0.214 | 0.198 | 0.173 | 1.0000 |
| phaA7 | 2 | 14 | 0.286 | 0.254 | 0.215 | 1.0000 |
| phaG1 | 3 | 14 | 0.714 | 0.521 | 0.407 | 0.1239 |
| phaJ5 | 2 | 14 | 0.357 | 0.304 | 0.250 | 1.0000 |
| phaJ8 | 2 | 14 | 0.857 | 0.508 | 0.370 | 0.0229 |
| phaJ9 | 3 | 14 | 0.286 | 0.265 | 0.240 | 1.0000 |
| Average | 2.4091 | - | 0.6235 | 0.4545 | 0.3640 | - |

**Table S6a** Genotyping results of 33 polymorphic microsatellite loci database of Laojunshan Sichuan Hill Partridge

| Sample | pha1a | pha1b | pha2a | pha2b | pha9a | pha9b | pha14a | pha14b | pha18a | pha18b | pha35a | pha35b | pha49a | pha49b | pha50a | pha50b |
| --- | --- | --- | --- | --- | --- | --- | --- | --- | --- | --- | --- | --- | --- | --- | --- | --- |
| RUF295 | 164 | 164 | 146 | 146 | 206 | 206 | 172 | 172 | 110 | 110 | 189 | 189 | 185 | 187 | 188 | 188 |
| RUF296 | 164 | 164 | 146 | 146 | 202 | 206 | 172 | 172 | 110 | 110 | 189 | 193 | 185 | 187 | 188 | 188 |
| RUF297 | 164 | 164 | 146 | 146 | 206 | 206 | 172 | 172 | 110 | 110 | 189 | 189 | 185 | 187 | 188 | 188 |
| RUF299 | 164 | 164 | 146 | 158 | 202 | 202 | 172 | 172 | 106 | 106 | 189 | 207 | 185 | 183 | 186 | 190 |
| RUF300 | 164 | 168 | 158 | 158 | 194 | 194 | 164 | 164 | 106 | 106 | 207 | 207 | 185 | 187 | 186 | 190 |
| RUF304 | 164 | 168 | 146 | 146 | 202 | 202 | 172 | 172 | 110 | 110 | 189 | 193 | 185 | 187 | 186 | 188 |
| RUF306 | 164 | 164 | 146 | 146 | 202 | 202 | 172 | 172 | 110 | 110 | 189 | 195 | 185 | 187 | 188 | 188 |
| RUF307 | 164 | 164 | 146 | 146 | 202 | 202 | 172 | 172 | 110 | 110 | 189 | 189 | 185 | 187 | 188 | 188 |
| RUF308 | 164 | 164 | 146 | 146 | 202 | 202 | 172 | 172 | 110 | 110 | 193 | 193 | 185 | 187 | 188 | 188 |
| RUF309 | 164 | 164 | 146 | 146 | 202 | 206 | 172 | 172 | 110 | 110 | 189 | 193 | 185 | 183 | 188 | 188 |
| RUF311 | 164 | 164 | 146 | 146 | 202 | 202 | 172 | 172 | 110 | 110 | 189 | 193 | 185 | 183 | 186 | 188 |
| RUF312 | 164 | 164 | 146 | 146 | 202 | 202 | 172 | 172 | 110 | 110 | 189 | 189 | 185 | 187 | 188 | 188 |
| RUF313 | 164 | 168 | 146 | 146 | 202 | 202 | 172 | 172 | 110 | 110 | 189 | 193 | 185 | 187 | 188 | 188 |
| RUF314 | 164 | 168 | 146 | 146 | 202 | 202 | 172 | 172 | 110 | 110 | 189 | 193 | 185 | 187 | 186 | 188 |
| RUF315 | 164 | 164 | 146 | 146 | 202 | 206 | 172 | 172 | 110 | 110 | 189 | 193 | 185 | 183 | 186 | 188 |
| RUF316 | 164 | 164 | 146 | 146 | 202 | 202 | 172 | 172 | 110 | 110 | 189 | 193 | 185 | 183 | 188 | 188 |
| RUF317 | 164 | 164 | 146 | 146 | 202 | 202 | 172 | 172 | 110 | 110 | 189 | 193 | 185 | 187 | 188 | 188 |
| RUF318 | 164 | 168 | 146 | 146 | 202 | 202 | 172 | 172 | 110 | 110 | 189 | 195 | 185 | 187 | 188 | 188 |
| RUF319 | 164 | 164 | 146 | 146 | 202 | 202 | 172 | 172 | 110 | 110 | 189 | 189 | 185 | 187 | 188 | 188 |
| RUF320 | 164 | 160 | 146 | 146 | 202 | 202 | 172 | 172 | 110 | 110 | 189 | 193 | 185 | 185 | 188 | 188 |
| RUF321 | 164 | 164 | 146 | 146 | 202 | 206 | 172 | 172 | 110 | 110 | 189 | 189 | 185 | 187 | 186 | 188 |

Continued Supplementary material

| Sample | pha51a | pha51b | pha53a | pha53b | pha55a | pha55b | pha57a | pha57b | pha58a | pha58b | pha62a | pha62b | pha69a | pha69b | pha71a | pha71b |
| --- | --- | --- | --- | --- | --- | --- | --- | --- | --- | --- | --- | --- | --- | --- | --- | --- |
| RUF295 | 183 | 183 | 164 | 164 | 119 | 121 | 195 | 197 | 147 | 147 | 190 | 190 | 177 | 177 | 159 | 161 |
| RUF296 | 183 | 183 | 164 | 166 | 119 | 121 | 195 | 197 | 147 | 147 | 190 | 190 | 177 | 179 | 159 | 161 |
| RUF297 | 183 | 183 | 164 | 166 | 119 | 121 | 195 | 197 | 147 | 147 | 190 | 190 | 177 | 179 | 159 | 161 |
| RUF299 | 173 | 173 | 164 | 164 | 117 | 121 | 189 | 197 | 147 | 147 | 190 | 198 | 177 | 179 | 157 | 157 |
| RUF300 | 173 | 173 | 164 | 166 | 117 | 117 | 189 | 197 | 147 | 147 | 198 | 200 | 177 | 179 | 157 | 157 |
| RUF304 | 181 | 183 | 164 | 164 | 119 | 121 | 195 | 197 | 147 | 147 | 190 | 190 | 177 | 179 | 159 | 161 |
| RUF306 | 183 | 183 | 164 | 166 | 119 | 121 | 197 | 197 | 147 | 147 | 190 | 190 | 177 | 179 | 159 | 161 |
| RUF307 | 181 | 183 | 164 | 164 | 119 | 121 | 195 | 197 | 147 | 147 | 190 | 190 | 177 | 179 | 159 | 161 |
| RUF308 | 181 | 183 | 164 | 164 | 119 | 121 | 197 | 197 | 147 | 147 | 190 | 190 | 177 | 179 | 159 | 161 |
| RUF309 | 183 | 183 | 164 | 164 | 119 | 121 | 195 | 197 | 161 | 161 | 190 | 190 | 177 | 179 | 159 | 161 |
| RUF311 | 183 | 183 | 164 | 166 | 119 | 121 | 195 | 197 | 147 | 147 | 188 | 190 | 177 | 179 | 159 | 161 |
| RUF312 | 181 | 183 | 164 | 166 | 119 | 121 | 195 | 197 | 147 | 147 | 190 | 190 | 177 | 179 | 159 | 161 |
| RUF313 | 181 | 183 | 164 | 166 | 119 | 121 | 195 | 197 | 147 | 147 | 190 | 190 | 177 | 179 | 159 | 161 |
| RUF314 | 183 | 183 | 164 | 166 | 119 | 121 | 195 | 197 | 147 | 147 | 190 | 190 | 177 | 179 | 159 | 161 |
| RUF315 | 183 | 183 | 164 | 164 | 119 | 121 | 197 | 197 | 147 | 147 | 190 | 190 | 177 | 179 | 159 | 161 |
| RUF316 | 183 | 183 | 164 | 164 | 119 | 121 | 195 | 197 | 147 | 147 | 190 | 190 | 177 | 179 | 159 | 161 |
| RUF317 | 183 | 183 | 164 | 164 | 119 | 121 | 195 | 197 | 147 | 147 | 188 | 190 | 177 | 177 | 159 | 161 |
| RUF318 | 181 | 183 | 164 | 166 | 119 | 121 | 195 | 197 | 147 | 147 | 190 | 190 | 177 | 179 | 159 | 161 |
| RUF319 | 181 | 183 | 164 | 166 | 119 | 121 | 197 | 197 | 147 | 147 | 190 | 190 | 177 | 177 | 159 | 161 |
| RUF320 | 181 | 183 | 164 | 166 | 119 | 121 | 195 | 197 | 147 | 147 | 190 | 190 | 177 | 179 | 159 | 161 |
| RUF321 | 181 | 183 | 164 | 166 | 119 | 121 | 195 | 197 | 147 | 147 | 190 | 190 | 177 | 179 | 159 | 161 |

Continued Supplementary material

| Sample | pha77a | pha77b | pha79a | pha79b | pha80a | pha80b | pha85a | pha85b | pha87a | pha87b | pha30a | pha30b | phaA1a | phaA1b | phaA3a | phaA3b |
| --- | --- | --- | --- | --- | --- | --- | --- | --- | --- | --- | --- | --- | --- | --- | --- | --- |
| RUF295 | 154 | 156 | 154 | 156 | 176 | 176 | 195 | 197 | 156 | 156 | 158 | 158 | 110 | 113 | 194 | 194 |
| RUF296 | 156 | 156 | 154 | 156 | 176 | 176 | 195 | 197 | 156 | 156 | 158 | 161 | 110 | 110 | 194 | 194 |
| RUF297 | 154 | 156 | 154 | 156 | 176 | 176 | 195 | 197 | 156 | 156 | 158 | 161 | 110 | 113 | 194 | 194 |
| RUF299 | 156 | 156 | 158 | 158 | 174 | 176 | 193 | 197 | 158 | 158 | 158 | 158 | 95 | 95 | 194 | 203 |
| RUF300 | 150 | 156 | 158 | 158 | 176 | 176 | 189 | 191 | 158 | 158 | 158 | 158 | 95 | 95 | 194 | 203 |
| RUF304 | 156 | 156 | 154 | 156 | 176 | 176 | 195 | 197 | 154 | 156 | 158 | 158 | 110 | 113 | 194 | 194 |
| RUF306 | 156 | 156 | 152 | 154 | 176 | 176 | 195 | 197 | 154 | 156 | 158 | 161 | 110 | 113 | 194 | 194 |
| RUF307 | 154 | 156 | 154 | 156 | 176 | 176 | 193 | 197 | 154 | 156 | 158 | 158 | 110 | 110 | 194 | 203 |
| RUF308 | 156 | 156 | 154 | 156 | 176 | 176 | 195 | 197 | 156 | 156 | 158 | 158 | 110 | 113 | 203 | 203 |
| RUF309 | 154 | 156 | 154 | 156 | 176 | 176 | 195 | 197 | 154 | 156 | 158 | 158 | 110 | 110 | 194 | 194 |
| RUF311 | 154 | 156 | 154 | 156 | 176 | 176 | 193 | 197 | 154 | 156 | 158 | 158 | 110 | 113 | 194 | 194 |
| RUF312 | 154 | 156 | 154 | 156 | 176 | 176 | 193 | 197 | 154 | 156 | 158 | 161 | 110 | 113 | 194 | 203 |
| RUF313 | 156 | 156 | 152 | 154 | 176 | 176 | 195 | 197 | 154 | 156 | 158 | 158 | 110 | 110 | 194 | 203 |
| RUF314 | 154 | 156 | 152 | 154 | 176 | 176 | 193 | 197 | 156 | 156 | 158 | 158 | 113 | 113 | 194 | 194 |
| RUF315 | 154 | 156 | 154 | 156 | 176 | 176 | 193 | 197 | 156 | 156 | 158 | 158 | 113 | 113 | 194 | 194 |
| RUF316 | 154 | 156 | 154 | 156 | 176 | 176 | 193 | 197 | 154 | 156 | 158 | 158 | 113 | 113 | 194 | 194 |
| RUF317 | 154 | 156 | 152 | 154 | 176 | 176 | 195 | 197 | 154 | 156 | 158 | 161 | 113 | 113 | 194 | 194 |
| RUF318 | 154 | 156 | 154 | 156 | 176 | 176 | 193 | 197 | 154 | 156 | 158 | 158 | 110 | 110 | 194 | 194 |
| RUF319 | 156 | 156 | 154 | 156 | 176 | 176 | 195 | 197 | 156 | 156 | 158 | 158 | 110 | 110 | 203 | 203 |
| RUF320 | 156 | 156 | 154 | 156 | 176 | 176 | 195 | 197 | 154 | 156 | 158 | 158 | 110 | 113 | 194 | 194 |
| RUF321 | 156 | 156 | 154 | 156 | 174 | 176 | 195 | 197 | 154 | 156 | 158 | 158 | 110 | 113 | 194 | 194 |

Continued Supplementary material

| Sample | phaA4a | phaA4b | phaA6a | phaA6b | phaA7a | phaA7b | phaG1a | phaG1b | phaG4a | phaG4b | phaG5a | phaG5b | phaJ5a | phaJ5b | phaJ6a | phaJ6b | phaJ8a | phaJ8b |
| --- | --- | --- | --- | --- | --- | --- | --- | --- | --- | --- | --- | --- | --- | --- | --- | --- | --- | --- |
| RUF295 | 176 | 176 | 174 | 174 | 127 | 130 | 171 | 177 | 192 | 192 | 128 | 128 | 141 | 150 | 111 | 114 | 164 | 167 |
| RUF296 | 176 | 176 | 174 | 177 | 130 | 130 | 177 | 177 | 192 | 192 | 128 | 128 | 141 | 150 | 111 | 114 | 164 | 167 |
| RUF297 | 176 | 176 | 174 | 174 | 130 | 130 | 177 | 177 | 192 | 192 | 128 | 128 | 141 | 141 | 111 | 114 | 164 | 167 |
| RUF299 | 167 | 176 | 174 | 177 | 130 | 130 | 177 | 177 | 198 | 192 | 128 | 137 | 138 | 138 | 111 | 111 | 164 | 167 |
| RUF300 | 167 | 167 | 174 | 177 | 130 | 130 | 183 | 177 | 198 | 201 | 137 | 137 | 138 | 138 | 111 | 111 | 164 | 167 |
| RUF304 | 176 | 176 | 174 | 177 | 127 | 130 | 177 | 177 | 192 | 192 | 128 | 128 | 141 | 150 | 111 | 111 | 164 | 167 |
| RUF306 | 176 | 176 | 174 | 177 | 127 | 130 | 171 | 177 | 192 | 192 | 128 | 128 | 141 | 141 | 111 | 114 | 167 | 167 |
| RUF307 | 176 | 176 | 174 | 177 | 127 | 130 | 177 | 177 | 192 | 192 | 128 | 128 | 141 | 150 | 111 | 114 | 164 | 167 |
| RUF308 | 176 | 176 | 174 | 174 | 127 | 130 | 177 | 177 | 192 | 192 | 128 | 128 | 141 | 150 | 111 | 114 | 164 | 167 |
| RUF309 | 176 | 176 | 174 | 174 | 127 | 130 | 177 | 177 | 192 | 192 | 128 | 128 | 141 | 129 | 111 | 111 | 164 | 167 |
| RUF311 | 176 | 176 | 171 | 174 | 127 | 130 | 177 | 177 | 192 | 192 | 128 | 128 | 141 | 150 | 111 | 111 | 164 | 167 |
| RUF312 | 176 | 176 | 174 | 177 | 127 | 130 | 177 | 177 | 192 | 192 | 128 | 128 | 141 | 150 | 111 | 111 | 164 | 167 |
| RUF313 | 176 | 176 | 174 | 177 | 130 | 130 | 177 | 177 | 192 | 192 | 128 | 128 | 141 | 141 | 111 | 114 | 167 | 167 |
| RUF314 | 176 | 176 | 168 | 168 | 130 | 130 | 177 | 177 | 192 | 192 | 128 | 128 | 141 | 141 | 111 | 114 | 164 | 167 |
| RUF315 | 176 | 176 | 174 | 174 | 127 | 130 | 171 | 177 | 192 | 192 | 128 | 128 | 141 | 150 | 111 | 114 | 164 | 167 |
| RUF316 | 176 | 176 | 174 | 177 | 130 | 130 | 177 | 177 | 192 | 192 | 128 | 128 | 141 | 150 | 111 | 111 | 164 | 167 |
| RUF317 | 176 | 176 | 171 | 177 | 130 | 130 | 177 | 177 | 192 | 192 | 128 | 128 | 141 | 150 | 111 | 111 | 164 | 167 |
| RUF318 | 176 | 176 | 174 | 177 | 127 | 130 | 177 | 177 | 192 | 192 | 128 | 128 | 141 | 150 | 111 | 111 | 164 | 167 |
| RUF319 | 176 | 176 | 174 | 177 | 130 | 130 | 177 | 177 | 192 | 192 | 128 | 128 | 141 | 150 | 111 | 111 | 167 | 167 |
| RUF320 | 176 | 176 | 174 | 174 | 130 | 130 | 177 | 177 | 192 | 192 | 128 | 128 | 141 | 150 | 111 | 114 | 164 | 167 |
| RUF321 | 176 | 176 | 174 | 174 | 127 | 130 | 177 | 177 | 192 | 192 | 128 | 128 | 141 | 141 | 111 | 111 | 164 | 167 |

**Table S6b** Genotyping results of 24 Polymorphic microsatellite loci database of Pamuling Blood Pheasant

| Sample | pha1a | pha1b | pha16a | pha16b | pha49a | pha49b | pha50a | pha50b | pha51a | pha51b | pha53a | pha53b | pha55a | pha55b | pha57a | pha57b |
| --- | --- | --- | --- | --- | --- | --- | --- | --- | --- | --- | --- | --- | --- | --- | --- | --- |
| ITH209 | 168 | 172 | 120 | 124 | 185 | 185 | 190 | 192 | 181 | 183 | 166 | 168 | 117 | 119 | 191 | 193 |
| ITH242 | 168 | 168 | 120 | 124 | 183 | 185 | 192 | 192 | 181 | 185 | 166 | 166 | 117 | 119 | 191 | 193 |
| ITH243 | 168 | 172 | 124 | 124 | 185 | 185 | 196 | 200 | 177 | 185 | 166 | 168 | 117 | 119 | 191 | 193 |
| ITH244 | 168 | 168 | 120 | 124 | 185 | 185 | 192 | 192 | 181 | 185 | 166 | 168 | 117 | 119 | 191 | 193 |
| ITH246 | 168 | 172 | 120 | 124 | 185 | 185 | 190 | 192 | 181 | 185 | 166 | 168 | 117 | 119 | 191 | 193 |
| ITH248 | 164 | 172 | 120 | 124 | 185 | 189 | 188 | 192 | 179 | 181 | 166 | 168 | 117 | 119 | 191 | 193 |
| ITH249 | 168 | 168 | 124 | 124 | 185 | 185 | 190 | 192 | 179 | 181 | 166 | 168 | 117 | 119 | 189 | 191 |
| ITH250 | 164 | 172 | 124 | 124 | 185 | 185 | 192 | 196 | 179 | 181 | 166 | 166 | 117 | 119 | 189 | 191 |
| ITH251 | 168 | 172 | 120 | 124 | 185 | 185 | 190 | 192 | 181 | 183 | 166 | 168 | 117 | 119 | 191 | 193 |
| ITH252 | 168 | 172 | 120 | 124 | 185 | 185 | 190 | 192 | 181 | 185 | 166 | 166 | 119 | 119 | 191 | 193 |
| ITH253 | 164 | 168 | 120 | 124 | 185 | 185 | 190 | 192 | 181 | 185 | 166 | 168 | 117 | 119 | 191 | 193 |
| ITH254 | 168 | 172 | 124 | 124 | 185 | 185 | 196 | 204 | 181 | 185 | 166 | 168 | 117 | 119 | 191 | 193 |
| ITH255 | 168 | 172 | 124 | 124 | 185 | 185 | 190 | 192 | 179 | 181 | 166 | 166 | 117 | 119 | 191 | 193 |
| ITH256 | 168 | 172 | 120 | 124 | 185 | 185 | 190 | 192 | 181 | 183 | 166 | 168 | 119 | 119 | 191 | 193 |
| ITH257 | 168 | 172 | 120 | 124 | 185 | 185 | 190 | 192 | 181 | 185 | 166 | 168 | 117 | 119 | 191 | 193 |
| ITH258 | 168 | 172 | 124 | 124 | 185 | 185 | 192 | 202 | 179 | 181 | 166 | 168 | 117 | 119 | 191 | 193 |
| ITH259 | 168 | 172 | 124 | 124 | 185 | 185 | 192 | 196 | 181 | 185 | 166 | 168 | 117 | 119 | 191 | 193 |
| ITH260 | 164 | 172 | 124 | 124 | 185 | 189 | 190 | 192 | 183 | 185 | 166 | 168 | 117 | 119 | 193 | 193 |
| ITH261 | 168 | 172 | 124 | 124 | 185 | 185 | 192 | 196 | 181 | 185 | 166 | 168 | 117 | 119 | 191 | 193 |

Continued Supplementary material

| Sample | pha60a | pha60b | pha64a | pha64b | pha69a | pha69b | pha70a | pha70b | pha77a | pha77b | pha79a | pha79b | pha80a | pha80b | pha85a | pha85b |
| --- | --- | --- | --- | --- | --- | --- | --- | --- | --- | --- | --- | --- | --- | --- | --- | --- |
| ITH209 | 105 | 107 | 177 | 179 | 177 | 179 | 118 | 120 | 158 | 160 | 152 | 154 | 174 | 176 | 195 | 197 |
| ITH242 | 105 | 107 | 179 | 179 | 179 | 179 | 118 | 120 | 158 | 160 | 150 | 152 | 176 | 176 | 195 | 197 |
| ITH243 | 105 | 107 | 179 | 179 | 179 | 179 | 118 | 118 | 158 | 160 | 152 | 154 | 176 | 176 | 195 | 197 |
| ITH244 | 105 | 107 | 177 | 179 | 179 | 179 | 118 | 118 | 158 | 160 | 150 | 152 | 174 | 176 | 195 | 197 |
| ITH246 | 105 | 107 | 179 | 179 | 177 | 179 | 118 | 118 | 158 | 160 | 150 | 152 | 174 | 176 | 195 | 197 |
| ITH248 | 105 | 107 | 179 | 179 | 179 | 179 | 118 | 120 | 158 | 160 | 152 | 154 | 176 | 176 | 195 | 197 |
| ITH249 | 105 | 107 | 179 | 181 | 177 | 177 | 118 | 118 | 162 | 160 | 152 | 154 | 176 | 176 | 195 | 197 |
| ITH250 | 105 | 107 | 179 | 179 | 179 | 179 | 118 | 118 | 162 | 160 | 150 | 152 | 176 | 176 | 195 | 197 |
| ITH251 | 105 | 107 | 179 | 179 | 177 | 179 | 118 | 118 | 158 | 160 | 152 | 154 | 176 | 176 | 195 | 197 |
| ITH252 | 105 | 107 | 179 | 179 | 179 | 179 | 118 | 120 | 162 | 160 | 150 | 152 | 176 | 176 | 195 | 197 |
| ITH253 | 109 | 107 | 179 | 181 | 177 | 177 | 118 | 118 | 158 | 160 | 150 | 152 | 176 | 176 | 195 | 197 |
| ITH254 | 109 | 111 | 177 | 179 | 177 | 179 | 118 | 118 | 158 | 160 | 152 | 154 | 174 | 176 | 197 | 197 |
| ITH255 | 109 | 111 | 179 | 181 | 179 | 179 | 118 | 118 | 158 | 160 | 152 | 152 | 174 | 176 | 195 | 197 |
| ITH256 | 105 | 107 | 179 | 179 | 177 | 179 | 118 | 120 | 158 | 160 | 152 | 154 | 174 | 176 | 197 | 197 |
| ITH257 | 105 | 107 | 179 | 179 | 177 | 179 | 118 | 118 | 158 | 160 | 150 | 152 | 174 | 176 | 195 | 197 |
| ITH258 | 105 | 103 | 177 | 179 | 179 | 179 | 118 | 118 | 158 | 160 | 150 | 152 | 174 | 176 | 195 | 197 |
| ITH259 | 105 | 103 | 179 | 179 | 177 | 179 | 118 | 120 | 158 | 160 | 150 | 152 | 174 | 176 | 197 | 197 |
| ITH260 | 105 | 107 | 179 | 179 | 179 | 179 | 118 | 120 | 158 | 160 | 150 | 152 | 176 | 176 | 197 | 197 |
| ITH261 | 105 | 107 | 179 | 181 | 177 | 179 | 118 | 120 | 158 | 160 | 150 | 152 | 174 | 176 | 195 | 197 |

Continued Supplementary material

| Sample | pha87a | pha87b | phaA1a | phaA1b | phaA3a | phaA3b | phaA7a | phaA7b | phaG1a | phaG1b | phaG5a | phaG5b | phaJ8a | phaJ8b | phaK2a | phaK2b |
| --- | --- | --- | --- | --- | --- | --- | --- | --- | --- | --- | --- | --- | --- | --- | --- | --- |
| ITH209 | 152 | 154 | 119 | 119 | 203 | 203 | 127 | 130 | 107 | 107 | 131 | 134 | 161 | 164 | 197 | 197 |
| ITH242 | 152 | 154 | 119 | 119 | 203 | 203 | 127 | 130 | 107 | 107 | 131 | 131 | 161 | 164 | 197 | 197 |
| ITH243 | 154 | 154 | 119 | 119 | 203 | 203 | 127 | 130 | 107 | 107 | 131 | 131 | 161 | 164 | 197 | 197 |
| ITH244 | 154 | 154 | 119 | 119 | 203 | 203 | 127 | 130 | 107 | 107 | 131 | 131 | 161 | 164 | 197 | 197 |
| ITH246 | 152 | 154 | 119 | 119 | 203 | 203 | 127 | 130 | 104 | 107 | 131 | 131 | 161 | 164 | 197 | 200 |
| ITH248 | 154 | 154 | 119 | 119 | 203 | 203 | 130 | 130 | 107 | 107 | 131 | 134 | 161 | 164 | 197 | 197 |
| ITH249 | 152 | 154 | 119 | 119 | 203 | 203 | 130 | 130 | 107 | 107 | 131 | 134 | 161 | 164 | 197 | 197 |
| ITH250 | 154 | 154 | 119 | 122 | 191 | 203 | 130 | 130 | 107 | 107 | 131 | 131 | 161 | 164 | 197 | 197 |
| ITH251 | 154 | 154 | 119 | 119 | 203 | 203 | 130 | 130 | 107 | 107 | 131 | 131 | 161 | 164 | 197 | 197 |
| ITH252 | 152 | 154 | 119 | 119 | 203 | 203 | 127 | 130 | 104 | 107 | 131 | 131 | 161 | 164 | 197 | 197 |
| ITH253 | 152 | 154 | 119 | 122 | 194 | 203 | 127 | 130 | 104 | 107 | 131 | 134 | 161 | 164 | 197 | 197 |
| ITH254 | 152 | 154 | 119 | 122 | 203 | 203 | 127 | 130 | 104 | 107 | 131 | 134 | 161 | 164 | 197 | 200 |
| ITH255 | 152 | 154 | 119 | 119 | 203 | 203 | 127 | 130 | 107 | 107 | 131 | 134 | 161 | 164 | 197 | 200 |
| ITH256 | 154 | 154 | 119 | 119 | 203 | 203 | 127 | 130 | 107 | 107 | 131 | 134 | 161 | 164 | 197 | 197 |
| ITH257 | 152 | 154 | 119 | 119 | 203 | 203 | 130 | 130 | 107 | 107 | 131 | 131 | 164 | 164 | 197 | 197 |
| ITH258 | 152 | 154 | 119 | 119 | 194 | 194 | 127 | 130 | 107 | 107 | 131 | 134 | 161 | 164 | 197 | 197 |
| ITH259 | 152 | 154 | 119 | 119 | 203 | 203 | 127 | 130 | 107 | 107 | 131 | 131 | 161 | 164 | 197 | 197 |
| ITH260 | 154 | 154 | 119 | 119 | 194 | 203 | 127 | 130 | 107 | 107 | 131 | 134 | 161 | 164 | 197 | 200 |
| ITH261 | 152 | 154 | 119 | 119 | 203 | 203 | 127 | 130 | 107 | 107 | 131 | 134 | 161 | 164 | 197 | 197 |

**Table S6c** Genotyping results of 22 Polymorphic microsatellite loci database of Pamuling Buff-throated Partridge

| Sample | pha9a | pha9b | pha14a | pha14b | pha35a | pha35b | pha43a | pha43b | pha51a | pha51b | pha53a | pha53b | pha55a | pha55b | pha60a | pha60b |
| --- | --- | --- | --- | --- | --- | --- | --- | --- | --- | --- | --- | --- | --- | --- | --- | --- |
| TET139 | 202 | 202 | 172 | 172 | 191 | 193 | 193 | 195 | 179 | 183 | 174 | 178 | 121 | 123 | 109 | 111 |
| TET162 | 202 | 202 | 172 | 172 | 193 | 195 | 193 | 195 | 179 | 179 | 176 | 178 | 121 | 121 | 109 | 111 |
| TET156 | 194 | 194 | 172 | 176 | 195 | 197 | 193 | 195 | 179 | 183 | 174 | 176 | 121 | 123 | 109 | 111 |
| TET152 | 194 | 194 | 172 | 176 | 195 | 197 | 193 | 195 | 179 | 183 | 176 | 178 | 121 | 123 | 109 | 111 |
| TET176 | 194 | 202 | 172 | 172 | 191 | 193 | 193 | 195 | 179 | 179 | 174 | 176 | 121 | 123 | 107 | 109 |
| TET164 | 202 | 202 | 172 | 172 | 191 | 193 | 191 | 195 | 179 | 179 | 174 | 178 | 121 | 123 | 107 | 109 |
| TET177 | 194 | 202 | 172 | 172 | 193 | 195 | 193 | 195 | 179 | 179 | 174 | 176 | 121 | 123 | 107 | 109 |
| TET173 | 194 | 202 | 172 | 176 | 195 | 197 | 193 | 195 | 179 | 183 | 174 | 176 | 121 | 123 | 109 | 111 |
| TET280 | 202 | 202 | 172 | 176 | 191 | 193 | 193 | 195 | 179 | 179 | 174 | 176 | 121 | 123 | 109 | 111 |
| TET174 | 202 | 202 | 172 | 172 | 193 | 195 | 193 | 195 | 179 | 179 | 174 | 176 | 121 | 121 | 109 | 111 |
| TET161 | 202 | 202 | 172 | 172 | 193 | 195 | 193 | 195 | 179 | 179 | 176 | 178 | 121 | 121 | 109 | 111 |
| TET151 | 194 | 202 | 172 | 172 | 191 | 193 | 193 | 195 | 179 | 183 | 174 | 176 | 121 | 123 | 107 | 109 |
| TET166 | 194 | 202 | 172 | 172 | 191 | 193 | 193 | 193 | 179 | 179 | 174 | 176 | 121 | 123 | 107 | 109 |
| TET137 | 194 | 194 | 172 | 172 | 193 | 195 | 193 | 195 | 179 | 179 | 174 | 174 | 121 | 121 | 107 | 109 |

Continued Supplementary material

| Sample | pha62a | pha62b | pha74a | pha74b | pha71a | pha71b | pha77a | pha77b | pha79a | pha79b | pha80a | pha80b | pha72a | pha72b | phaA1a | phaA1b |
| --- | --- | --- | --- | --- | --- | --- | --- | --- | --- | --- | --- | --- | --- | --- | --- | --- |
| TET139 | 190 | 190 | 198 | 198 | 165 | 167 | 154 | 156 | 154 | 154 | 174 | 176 | 136 | 136 | 113 | 113 |
| TET162 | 190 | 190 | 198 | 202 | 165 | 167 | 156 | 156 | 154 | 156 | 176 | 176 | 136 | 136 | 113 | 113 |
| TET156 | 190 | 190 | 198 | 198 | 165 | 167 | 154 | 156 | 154 | 156 | 174 | 176 | 134 | 136 | 113 | 116 |
| TET152 | 190 | 190 | 198 | 202 | 165 | 167 | 154 | 156 | 154 | 156 | 174 | 176 | 134 | 136 | 113 | 116 |
| TET176 | 190 | 192 | 198 | 202 | 165 | 167 | 154 | 156 | 154 | 152 | 174 | 176 | 134 | 136 | 113 | 116 |
| TET164 | 190 | 192 | 198 | 198 | 165 | 167 | 154 | 156 | 154 | 152 | 174 | 176 | 136 | 136 | 113 | 113 |
| TET177 | 190 | 190 | 198 | 198 | 165 | 167 | 154 | 156 | 154 | 152 | 174 | 176 | 134 | 136 | 113 | 116 |
| TET173 | 190 | 190 | 198 | 198 | 165 | 167 | 154 | 156 | 154 | 156 | 174 | 176 | 136 | 136 | 113 | 116 |
| TET280 | 190 | 190 | 198 | 198 | 165 | 167 | 154 | 156 | 154 | 152 | 174 | 176 | 136 | 136 | 113 | 116 |
| TET174 | 190 | 192 | 202 | 202 | 169 | 167 | 154 | 156 | 154 | 156 | 174 | 176 | 134 | 136 | 113 | 116 |
| TET161 | 190 | 192 | 198 | 202 | 169 | 167 | 154 | 156 | 154 | 152 | 174 | 176 | 136 | 136 | 113 | 113 |
| TET151 | 192 | 192 | 198 | 202 | 165 | 167 | 154 | 156 | 154 | 152 | 176 | 176 | 136 | 136 | 113 | 113 |
| TET166 | 190 | 192 | 198 | 202 | 165 | 167 | 154 | 156 | 154 | 152 | 174 | 176 | 136 | 136 | 113 | 113 |
| TET137 | 190 | 190 | 202 | 202 | 165 | 167 | 154 | 156 | 154 | 156 | 174 | 176 | 134 | 136 | 113 | 113 |

Continued Supplementary material

| Sample | phaA6a | phaA6b | phaA7a | phaA7b | phaG1a | phaG1b | phaJ5a | phaJ5b | phaJ8a | phaJ8b | phaJ9a | phaJ9b |
| --- | --- | --- | --- | --- | --- | --- | --- | --- | --- | --- | --- | --- |
| TET139 | 168 | 168 | 130 | 130 | 186 | 186 | 180 | 180 | 164 | 164 | 158 | 158 |
| TET162 | 168 | 168 | 130 | 130 | 186 | 186 | 180 | 180 | 161 | 164 | 158 | 161 |
| TET156 | 168 | 168 | 130 | 130 | 186 | 189 | 180 | 180 | 161 | 164 | 158 | 167 |
| TET152 | 168 | 168 | 127 | 130 | 186 | 189 | 171 | 180 | 164 | 164 | 158 | 158 |
| TET176 | 168 | 168 | 127 | 130 | 186 | 189 | 180 | 180 | 161 | 164 | 158 | 158 |
| TET164 | 162 | 168 | 130 | 130 | 186 | 189 | 171 | 180 | 161 | 164 | 158 | 158 |
| TET177 | 168 | 168 | 127 | 130 | 186 | 189 | 171 | 180 | 161 | 164 | 158 | 158 |
| TET173 | 168 | 168 | 127 | 130 | 189 | 192 | 180 | 180 | 161 | 164 | 158 | 158 |
| TET280 | 168 | 168 | 130 | 130 | 186 | 186 | 171 | 180 | 161 | 164 | 158 | 158 |
| TET174 | 162 | 168 | 130 | 130 | 186 | 189 | 180 | 180 | 161 | 164 | 158 | 161 |
| TET161 | 168 | 168 | 130 | 130 | 186 | 189 | 180 | 180 | 161 | 164 | 158 | 158 |
| TET151 | 168 | 168 | 130 | 130 | 186 | 186 | 180 | 180 | 161 | 164 | 158 | 158 |
| TET166 | 162 | 168 | 130 | 130 | 186 | 189 | 180 | 180 | 161 | 164 | 158 | 158 |
| TET137 | 168 | 168 | 130 | 130 | 186 | 189 | 171 | 180 | 161 | 164 | 158 | 167 |
